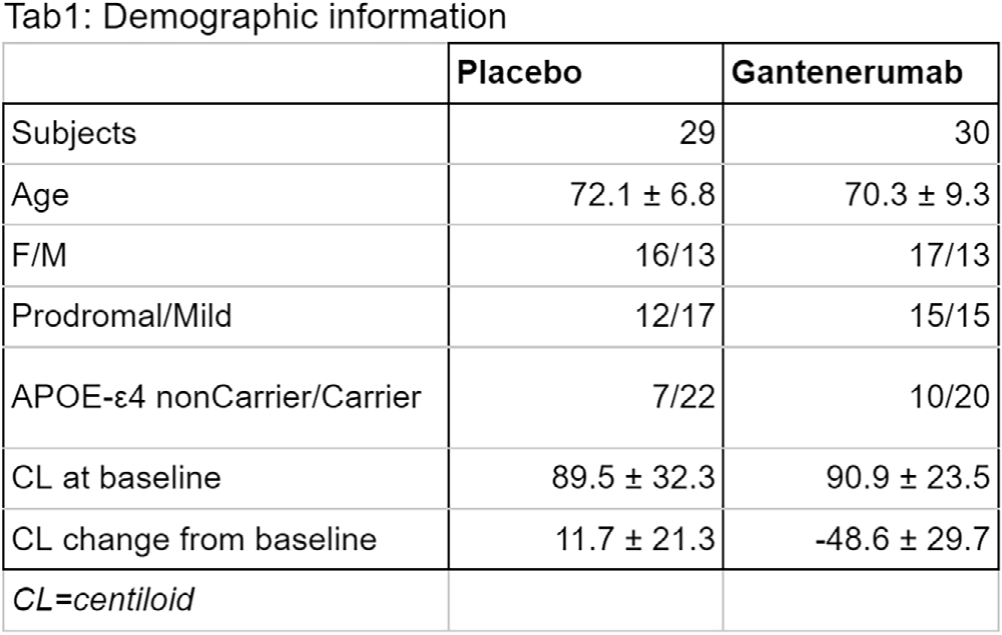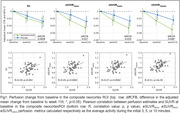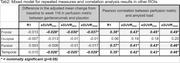# Longitudinal effects of gantenerumab treatment on amyloid‐PET‐derived perfusion estimates in an early AD population

**DOI:** 10.1002/alz.094958

**Published:** 2025-01-09

**Authors:** Erica Silvestri, Matteo Tonietto, Susanna Gobbi, Marzia Antonella Scelsi, Gregory Klein

**Affiliations:** ^1^ Roche Pharma Research and Early Development, Neuroscience and Rare Diseases Biomarkers, F. Hoffmann‐La Roche Ltd., Basel Switzerland; ^2^ A4P Consulting Ltd., Sandwich United Kingdom; ^3^ Roche Products Ltd., Product Development Data Sciences, Welwyn Garden City United Kingdom

## Abstract

**Background:**

Amyloid PET imaging enables the simultaneous assessment of both perfusion and amyloid deposition in people with Alzheimer’s Disease (AD) in a single procedure. Perfusion can be derived from kinetic modeling of the full dynamic PET acquisition (i.e., relative blood flow ‐ *R1*), or more simply as ‘early‐frame’ Standardized Uptake Value Ratio (*eSUVR)*. However, simplified measures could be biased by the amyloid tracer binding to amyloid. Here, we 1) assessed the effect of gantenerumab, an investigational anti‐amyloid monoclonal antibody, on perfusion metrics; 2) tested if the presence of amyloid affects perfusion estimation.

**Method:**

Fifty‐nine participants underwent dynamic [18F]‐florbetaben PET imaging at baseline and week 116 in the GRADUATE I and II phase III trials of gantenerumab in early AD (NCT03444870, NCT03443973) (Tab. 1). A dual‐time‐window acquisition was employed (0‐30 and 90‐110 mins windows). Perfusion and amyloid load were estimated in: frontal, parietal, temporal, and occipital cortices, and composite neocortex region of interest (ROI), using the cerebellar gray matter as reference region. Perfusion was assessed as *R1* using the simplified reference tissue model; and as e*SUVR* calculating the average activity during the initial 3, 5, or 10 minutes. Amyloid load was computed from the late time‐window as *SUVR*. Differences in mean perfusion change from baseline between placebo and gantenerumab were tested using a mixed model for repeated measures adjusted for treatment and APOE‐ε4 status. Pearson correlation measured the relationship between amyloid load and perfusion at baseline. All analyses were exploratory in nature and no adjustment for multiplicity was applied.

**Result:**

No treatment effect was observed on *R1* in any of the ROIs considered. However, gantenerumab arm resulted in a greater perfusion decrease when using *eSUVR*, with greater effects observed with longer *eSUVR* windows. Yet, all perfusion estimates correlated positively with amyloid load, with the correlation strength increasing with longer *eSUVR* time windows (Fig. 1, Tab. 2).

**Conclusion:**

Perfusion significantly decreased in both arms, with *R1* showing no difference between the two. PET‐derived perfusion metrics correlate positively with amyloid load, indicating amyloid‐dependence. Although kinetic modeling mitigates this bias, it does not fully eliminate it, warranting careful interpretation of these metrics in anti‐amyloid treatment assessments.